# Survival predictor in patients with acute respiratory distress syndrome and diffuse alveolar damage undergoing open lung biopsy

**DOI:** 10.1371/journal.pone.0180018

**Published:** 2017-07-05

**Authors:** Kuo-Chin Kao, Chih-Hao Chang, Chen-Yiu Hung, Li-Chung Chiu, Chung-Chi Huang, Han-Chung Hu

**Affiliations:** 1 Department of Thoracic Medicine, Chang Gung Memorial Hospital, Chang Gung University College of Medicine, Taoyuan, Taiwan; 2 Department of Respiratory Therapy, Chang-Gung University College of Medicine, Taoyuan, Taiwan; Universitatsklinikum Freiburg, GERMANY

## Abstract

**Background:**

Diffuse alveolar damage (DAD) is a typical pathological finding of open lung biopsies in patients with acute respiratory distress syndrome (ARDS). Patients with ARDS and DAD have been reported to have a poorer prognosis than those without DAD. The aim of this study was to investigate the survival predictors in patients with ARDS and DAD.

**Methods:**

We retrospectively reviewed all ARDS patients who underwent an open lung biopsy which showed evidence of DAD from January 2006 to June 2015 at Chang Gung Memorial Hospital. Clinical data including baseline characteristics, medication, and survival outcomes were analyzed.

**Results:**

A total of 64 ARDS patients with DAD were eligible for analysis and divided into known etiology (n = 17, 26.6%) and unknown etiology groups (n = 47, 73.4%). There was no significant difference in hospital mortality rate between the two groups (71.9% vs. 70.6%, *p* = 0.890). Univariate logistic regression analysis revealed that sequential organ failure assessment (SOFA) score at the time of a diagnosis of ARDS, and SOFA score, PaO_2_/FiO_2_ ratio, and positive end expiratory pressure level when the biopsy was performed were associated with hospital mortality. Multivariate analysis showed that the SOFA score on the day of the biopsy was an independent predictor of hospital mortality (odds ratio 1.413, 95% confidence interval 1.127–1.772; *p* = 0.03). There were no significant differences in the use, dose, duration and timing from ARDS to glucocorticoid therapy between the survivors and nonsurvivors.

**Conclusion:**

For selected ARDS patients who underwent an open lung biopsy with pathological DAD, SOFA score was an independent predictor of hospital mortality.

## Introduction

Diffuse alveolar damage (DAD) is the pathological hallmark of acute respiratory distress syndrome (ARDS) and is characterized by hyaline membranes, lung edema, inflammation, hemorrhage and alveolar epithelial cell damage [1,2]. However, DAD is not included in the Berlin definition of ARDS because an open lung biopsy (OLB) may be associated with an increased risk of complications [3]. DAD has been reported in 56%-58% of ARDS patients undergoing an OLB and in 33%-45% of those with autopsy examinations [4–7]. The presence of DAD in ARDS patients has been reported to be associated with a greater severity of disease and to be an independent risk factor for mortality [5,7]. A recent meta-analysis found that ARDS patients with DAD were associated with a higher risk of mortality than those without DAD [8]. Regarding the high mortality rate in ARDS patients, DAD may represent a specific sub-phenotype for which an effective therapy is needed [9].

Acute interstitial pneumonia (AIP) is an uncommon disease and a distinct clinicopathologic entity, defined as the clinical diagnosis of ARDS of unknown etiology and a pathological finding of DAD on lung biopsy [10,11]. The clinical prognosis of AIP patients is poor with a reported mortality rate ranging from 13%-100% [12]. The most common therapy for AIP is glucocorticoids, however their efficacy remains inconclusive.

Glucocorticoid therapy for ARDS patients is controversial, and the available evidence is inconsistent [13–18]. The ARDS Network’s LaSRS study did not advise the routine use of methylprednisolone in patients with persistent ARDS, and suggested that it is harmful if started more than 14 days after the onset of the syndrome [14]. However, a recent meta-analysis study showed that prolonged glucocorticoid treatment can hasten the resolution of ARDS, improve clinical outcomes and decrease hospital mortality and the use of healthcare resources [18]. It would therefore be helpful to investigate the effect of glucocorticoid therapy in a more homogeneous subgroup of ARDS patients, such as those with pathologically confirmed DAD. The purpose of this study was to investigate the predictors of survival, including glucocorticoid response, in patients with ARDS and DAD undergoing an OLB.

## Materials and methods

We performed this retrospective chart review of all patients with ARDS who underwent an OLB from January 2006 to June 2015 at the Linkou Chang Gung Memorial Hospital. This study was approved and the need for informed consent was waived by the Institutional Review Board at Chang-Gung Medical Foundation (CGMH IRB No.103-4332B). All patients admitted to intensive care units (ICU) who met the criteria for ARDS according to the Berlin definition for ARDS were enrolled for screening [19]. Patients with pathological diagnoses of DAD were eligible for analysis. The microbiological examinations performed before OLB and indications for OLB have been previously described [5,20].

### Microbiological examinations and open lung biopsy

The results of cultures from sputum, tracheal aspirate, blood, and pleural effusion were recorded. All of the patients received fibrobronchoscopic examinations with bronchoalveolar lavage (BAL) examinations before the OLB procedure. The location for BAL sampling was determined based on the findings from chest X-rays or high-resolution computed tomography (HRCT) of the chest. The BAL samples were sent to the hospital’s microbiology and pathology laboratories for examinations for bacteria, fungi and viruses, in accordance with normal practice. The bacterial study included aerobic, anaerobic, Legionella, Mycoplasma pneumonia and Mycobacteria. Urinary antigen testing was performed to detect Legionella pneumophila and Streptococcus pneumoniae (BinaxNOW, Alere). The fungal analysis included Pneumocystis jirovecii, Candida culture and Aspergillosis antigen and culture. The BAL samples were subjected to Giemsa and Gomori methenamine silver staining or qualitative pneumocystis DNA for Pneumocystis jirovecii was detected using a polymerase chain reaction (PCR). Viruses were detected in the BAL samples by PCR and virus culture, including reverse-transcription PCR for influenza virus A and B, shell vial culture for cytomegalovirus (CMV), and virus culture for parainfluenza virus, herpes simplex virus, adenovirus, herpes simplex virus, respiratory syncytial virus, human metapneumovirus and enterovirus. Specimens were also sent for iron stain analysis and cytology. The BAL results were defined positive when at least one microorganism had grown to a concentration >10^4^ colony-forming units/mL.

An OLB was considered when ARDS was thought to be noninfectious and glucocorticoid therapy was possible, based on the presentations of a rapid progression, symmetric distribution of infiltrates on chest X ray, or predominant ground-glass attenuation on HRCT of the chest. Informed consent for the operation was obtained from each patient’s family before the procedure.

### Pathological diagnosis of diffuse alveolar damage

All of the pathology results were reviewed and confirmed by an experienced pulmonary pathologist. The pathological criteria for the diagnosis of DAD were the presence of pulmonary inflammatory infiltrates, hyaline membrane formation and at least one of the following: alveolar type I cell necrosis, intra-alveolar edema and alveolar type II cell proliferation progressively covering the denuded alveolar-capillary membrane, interstitial proliferation of myofibroblasts and fibroblasts, or organizing interstitial fibrosis [1,2].

### Classification, management and outcomes

The medical records of the ARDS patients with pathological findings of DAD on OLB were reviewed to identify the etiology of ARDS. ARDS of unknown etiology was defined if the patients had no underlying pulmonary disease and no clinical risk factors for ARDS such as pneumonia, sepsis, aspiration of gastric contents, inhalational injury, collagen disease, drug abuse, pancreatitis, burns, or shock. ARDS of known etiology was defined if the patients had underlying pulmonary diseases or clinical risk factors for ARDS.

The following detailed clinical data were recorded: age, gender, underlying diseases, Acute Physiology and Chronic Health Evaluation (APACHE) II score [21], sequential organ failure assessment (SOFA) score [22], PaO_2_/FiO_2_ ratio, positive-end expiratory pressure (PEEP), tidal volume, and medications such as neuromuscular blockade agents, sedatives and glucocorticoids. A low and high dose of glucocorticoids was defined as ≤ 2 mg/kg/day and > 2 mg/kg/day equivalent dose of methylprednisolone, respectively. Surgery-related complications (i.e., postoperative air leak, subcutaneous emphysema, pneumothorax, hemothorax, and wound infection) and survival outcomes were also recorded.

### Statistical analysis

Data were presented as mean ± standard deviation for continuous variables, and frequency and percentage for categorical variables. Risk factors for in-hospital mortality were analyzed using analysis. The variables significantly associated with the outcome in univariate analysis (*P*<0.2) were included in the multivariate analysis which involved multiple logistic regression based on the backward elimination of data. The Hosmer-Lemeshow goodness-of-fit test was used for calibration when evaluating the number of observed and predicted deaths in each group. All statistical analyses were performed using MedCalc, version 12.5 (MedCalc Software, Ostend, Belgium). Two-tailed p values < 0.05 were considered to be statistically significant.

## Results

**Fig 1:**
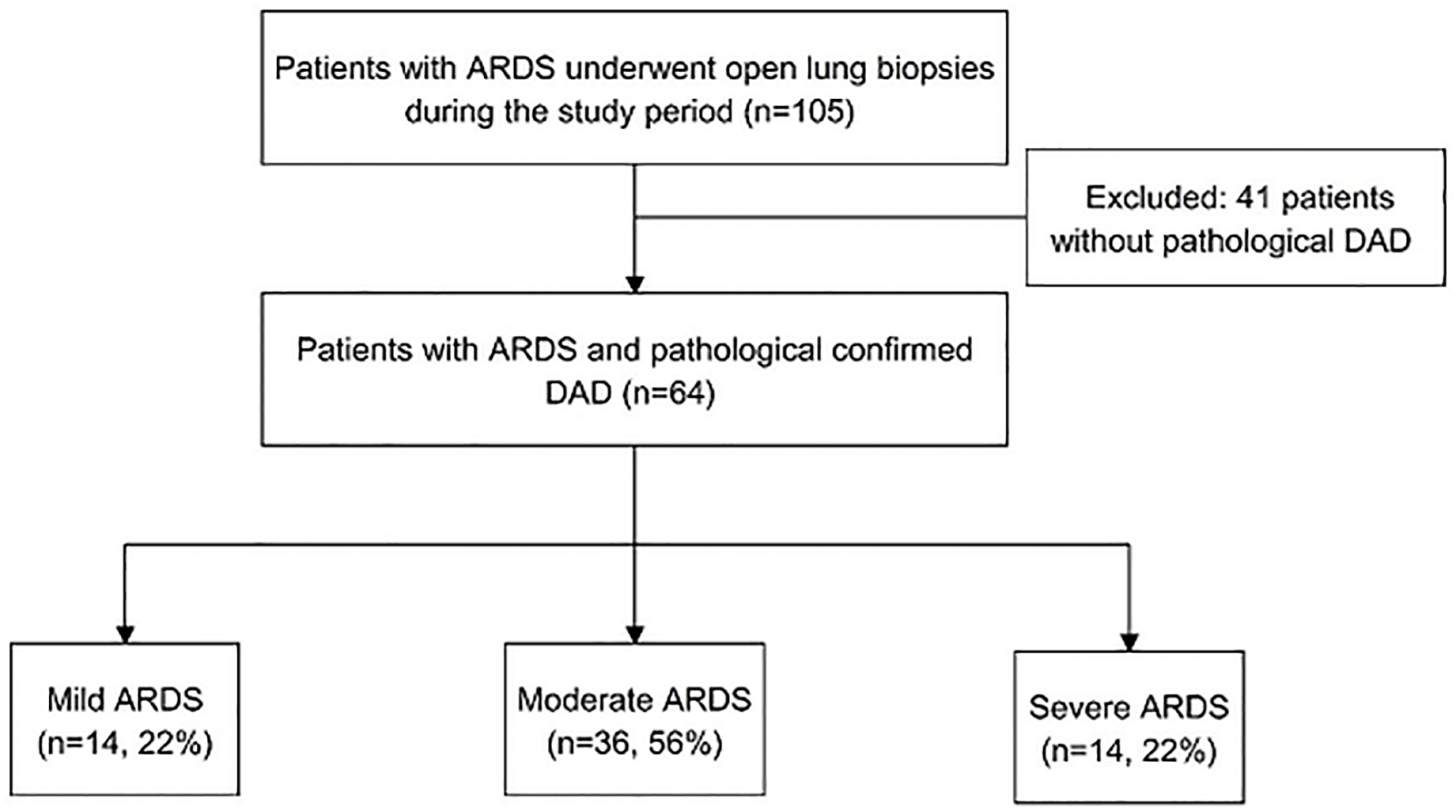
Flow chart for patients’ enrollment in this study. ARDS, acute respiratory distress syndrome, DAD diffuse alveolar damage.

In the study period, 105 patients were admitted to our ICUs with a diagnosis of ARDS who underwent OLB. Table 1 shows comparisons of clinical characteristics between the ARDS patients with and without DAD. The hospital mortality rate was higher in the patients with DAD than in those without DAD (71.9% vs. 43.9%; *P* = 0.004). Among these 105 patients, 64 (61%) with a pathological diagnosis of DAD were eligible for analysis (Fig 1). Of these 64 patients, most were classified as having moderate ARDS (n = 36, 56.3%). The hospital mortality rates for the mild, moderate and severe ARDS groups were 64.3%, 69.4% and 87.5%, respectively. The mean duration from the onset of ARDS to OLB was 8.6 ± 9.8 days. Biopsy specimens were obtained by video-assisted thoracoscopic surgery in 39 patients (61%), and by thoracotomy in 25 patients (39%). Of these 64 patients with a pathological diagnosis of DAD, 7 (10.9%) had surgical complications, including five with postoperative subcutaneous emphysema, one with pneumothorax and one with prolonged air leakage. The patient who was complicated by prolonged air leakage died.

**Table 1:** Demographics and clinical characteristics of with DAD and without DAD in ARDS patients undergoing open lung biopsy.

Characteristics	With DAD (n = 64)	Without DAD (n = 41)	*p* value
Age	57.6±16.9	56.9±16.2	0.819
Gender (male/female)	41/23	27/14	0.852
Immunocompromised (%)	26.6	24.4	0.805
APACHE II score	22.5±5.5	24.0±5.2	0.284
SOFA score, diagnosis day	6.3±3.7	5.8±2.5	0.461
PaO_2_/FiO_2_(mmHg), diagnosis day	148.1±60.8	134.6±57.8	0.258
SOFA score, biopsy day	7.2±3.8	6.7±2.7	0.466
PaO_2_/FiO_2_(mm Hg), biopsy day	160.5±70.6	135.1±61.1	0.061
PEEP (cm H_2_O), biopsy day	11.9±2.7	11.4±1.5	0.485
Tidal volume (ml), biopsy day	433.1±105.8	434.3±81.0	0.972
Surgical complication rate (%)	10.9	19.5	0.223
Hospital mortality	46 (71.9%)	18 (43.9%)	0.004*

All values are expressed as No. of patients (%) or mean ± standard deviation (SD).

*p <0.05: with DAD vs without DAD

DAD, diffuse alveolar damage; ARDS, Acute Respiratory Distress Syndrome; APACHE, Acute Physiology and Chronic Health Evaluation; SOFA, Sequential Organ Failure Assessment; PaO_2_, arterial partial pressure of oxygen; FiO_2_, fraction of inspired oxygen; PEEP, positive end-expiratory pressure.

Table 2 lists comparisons of the clinical characteristics between the survivors (n = 18, 28.1%) and nonsurvivors (n = 46, 71.9%). There was a statistically significant difference between the two groups in SOFA score on the day that ARDS was diagnosed, and the SOFA score, PaO_2_/FiO_2_ ratio, and PEEP level on the day the biopsy was performed. Regarding glucocorticoid treatment, there were no significant differences between the survivors and nonsurvivors in dosage, duration and time from onset of ARDS to glucocorticoid therapy. Multivariate logistic regression revealed that the SOFA score on the day the biopsy was performed was an independent predictor of hospital mortality (odds ratio: 1.413; 95% confidence interval: 1.127–1.772; *P* = 0.03) (Table 3).

**Table 2:** Demographics and clinical characteristics of survivors and non-survivors in ARDS patients with pathological DAD.

Characteristics	All patients (n = 64)	Survivors (n = 18)	Nonsurvivors (n = 46)	*p* value
Age	57.6±16.9	59.9±12.4	56.7±18.4	0.500
Gender (male/female)	41/23	9/9	32/14	0.142
Immunocompromised (%)	26.6	33.3	23.9	0.443
APACHE II score	22.5±5.5	21.1±4.9	22.8±5.6	0.477
SOFA score, diagnosis day	6.3±3.7	4.8±3.2	6.9±3.7	0.035*
PaO_2_/FiO_2_(mmHg), diagnosis day	148.1±60.8	150.7±59.3	147.1±62.0	0.836
SOFA score, biopsy day	7.2±3.8	4.6±2.9	8.2±3.6	<0.001*
PaO_2_/FiO_2_(mm Hg), biopsy day	160.5±70.6	194.9±82.8	146.8±60.8	0.013*
PEEP (cm H_2_O), biopsy day	12.2±3.0	10.9±3.0	12.7±2.8	0.031*
Tidal volume (ml), biopsy day	416.9±91.1	442.3±66.9	405.0±99.2	0.294
Surgical complication rate (%)	10.9	5.6	13.0	0.388
Medication use				
Neuromuscular blockade (%)	56.3	50.0	58.7	0.528
Sedatives (%)	62.5	61.1	63.0	0.886
Glucocorticoid, n (%)	79.7	16 (88.9)	35 (76.1)	0.252
Methylprednisolone equivalent dose (mg/kg/day)		3.56	3.85	0.780
Duration (days)		13.3±7.5	18.1±12.3	0.149
Time from ARDS onset (days)		6.2±7.9	7.3±8.7	0.660

All values are expressed as No. of patients (%) or mean ± standard deviation (SD).

*p <0.05: Survivors vs Nonsurvivors

ARDS, Acute Respiratory Distress Syndrome; APACHE, Acute Physiology and Chronic Health Evaluation; SOFA, Sequential Organ Failure Assessment; PaO_2_, arterial partial pressure of oxygen; FiO_2_, fraction of inspired oxygen; PEEP, positive end-expiratory pressure; DAD, diffuse alveolar damage

**Table 3:** Univariate and multivariate logistic regression analysis of clinical variables associated with hospital mortality in ARDS patients with pathologic DAD.

Parameter	Standard error	Odds ratios (95% CI)	*p* value
**Univariate analysis**			
Age	0.017	0.988 (0.956–1.022)	0.494
Female sex	0.570	0.438 (0.143–1.337)	0.147
SOFA score, diagnosis day	0.101	1.224 (1.004–1.492)	0.045*
SOFA score, biopsy day	0.114	1.424 (1.138–1.782)	0.002*
PaO_2_/FiO_2_(mmHg), biopsy day	0.004	0.990 (0.982–0.999)	0.023*
PEEP (cm H_2_O), biopsy day	0.103	1.238 (1.012–1.513)	0.038*
Steroid use	0.831	0.450 (0.088–2.293)	0.337
**Multivariate analysis**			
SOFA score, biopsy day	0.116	1.413 (1.127–1.772)	0.003*

*p <0.05

ARDS, Acute Respiratory Distress Syndrome; AIP, acute interstitial pneumonia; C.I., confidence interval; SOFA, Sequential Organ Failure Assessment; PaO_2_, arterial partial pressure of oxygen; FiO_2_, fraction of inspired oxygen; PEEP, positive end-expiratory pressure; DAD, diffuse alveolar damage. In multivariable logistic regression, the Hosmer–Lemeshow test results (χ2 = 5.382, degrees of freedom = 7, *p* = 0.613).

We then divided the 64 ARDS patients with DAD into an unknown etiology group (n = 17, 27%) and a known etiology group (n = 47, 73%) (Table 4). For the 47 patients with a known etiology, 17 had a specific pathological diagnosis of DAD, including infectious disease (n = 12), interstitial lung disease (n = 2), metastatic carcinoma (n = 2), and collagen vascular disease (n = 1). Another 30 patients had the following known risk factors for ARDS: pneumonia (n = 20), sepsis (n = 7), collagen vascular diseases (n = 1), drug abuse (n = 1), and pulmonary contusion (n = 1). The unknown etiology group had a lower percentage of male patients and a higher PaO_2_/FiO_2_ ratio on the day the biopsy was performed than the known etiology group (41% vs. 72%, *P* = 0.022; 202.3 ± 84.4 vs. 146.3 ± 60.0, *P* = 0.005). There were no significant differences in age, immune status, APACHE II score, SOFA score, PEEP, tidal volume, medications, surgical complication rate or survival outcomes between the two groups.

**Table 4:** Demographics and clinical characteristics of ARDS patients with pathological DAD.

Characteristics	All patients (n = 64)	Unknown etiology (n = 17)	Known etiology (n = 47)	*p* value
Age, years	57.6±16.9	62.5±14.2	55.9±17.6	0.171
Gender (male/female)	41/23	7/10	34/13	0.022*
Immunocompromised (%)	26.6	11.8	31.9	0.107
APACHE II score	22.5±5.5	21.3±8.1	22.9±4.5	0.476
SOFA score, diagnosis day	6.3±3.7	5.9±3.7	6.5±3.7	0.577
SOFA score, biopsy day	7.2±3.8	5.9±3.7	7.6±3.7	0.106
PaO_2_/FiO_2_ (mmHg), diagnosis day	148.1±60.8	169.4±64.2	140.4±58.3	0.092
PaO_2_/FiO_2_(mmHg), biopsy day	160.5±70.6	202.3±84.4	146.3±60.0	0.005*
PEEP (cm H_2_O), biopsy day	12.2±3.0	11.9±2.5	12.3±3.1	0.627
Tidal volume (ml), biopsy day	416.9±91.1	414.0±106.2	418.2±85.2	0.889
Surgical complication rate (%)	10.9	17.6	8.5	0.301
Medication use				
Neuromuscular blockade (%)	56.3	58.8	55.3	0.803
Sedatives (%)	62.5	64.7	61.7	0.826
Glucocorticoid (%)	79.7	76.5	80.9	0.700
ICU stay, days	24.7±18.8	18.0±9.4	27.1±20.7	0.085
Hospital stay, days	43.6±31.6	31.2±18.6	48.1±34.3	0.060
Hospital mortality rate (%)	71.9	70.6	72.3	0.890

All values are expressed as No. of patients (%) or mean ± standard deviation (SD).

*p <0.05: Unknown etiology vs Known etiology

ARDS, Acute Respiratory Distress Syndrome; APACHE, Acute Physiology and Chronic Health Evaluation; SOFA, Sequential Organ Failure Assessment; PaO_2_, arterial partial pressure of oxygen; FiO_2_, fraction of inspired oxygen; PEEP, positive end-expiratory pressure; DAD, diffuse alveolar damage.

## Discussion

DAD is the pathological hallmark of ARDS. In this study, DAD was found in 56%-58% of the ARDS patients undergoing OLB, and the presence of DAD was associated with a higher mortality rate. The major finding of this study is that the SOFA score on the day the biopsy was performed was an independent predictor of hospital mortality in ARDS patients undergoing OLB with pathological DAD.

Calfee et al. used latent class analysis to identify two subphenotypes of ARDS from two NHLBI ARDS randomized controlled trials [23]. They found that a hyperinflammatory phenotype with more severe inflammation, shock and significant metabolic acidosis led to a significantly worse clinical outcome. The other molecular phenotype involved direct lung injury, and was characterized by a higher level of surfactant protein D and lower level of angiopoietin-2, consistent with more severe lung epithelial injury and less severe endothelial injury [24]. More recently, a specific phenotype of ARDS characterized by DAD and a higher mortality rate has been identified [4–8]. In the present study we enrolled ARDS patients with DAD, and found that SOFA score, representing systemic inflammation or multiple organ damage, was an independent predictor of mortality. It is unclear whether the hyperinflammatory phenotype was more likely to be associated with DAD, SOFA score, or even mortality, as biomarkers were not available in this study.

The benefits and safety of OLB are controversial in patients in an ICU and in those requiring mechanical ventilation, even though its role has been established in the setting of interstitial lung disease [25]. In selected patients with ARDS, surgical complication rates of OLB have been reported to range from 17% to 39% [20,26,27]. Although the most common complications such as pneumothorax can be effectively treated and mortality from OLB is uncommon, the average surgical complication rate remains as high as 22% [28]. In this study, the overall surgical complication rate of OLB was 10.9%, and there seemed to be a higher trend in the nonsurvivors than in the survivors (13% vs. 5.6%, *P* = 0.388). Nevertheless, it should be noted that patients must be very carefully selected when considering an OLB due to the high rates of morbidity and mortality in ARDS patients. Identifying DAD with less invasive procedures with lower complication rates and obtaining adequate specimens for pathological examinations are important clinical and practical issue, especially in ARDS patients. Given that transbronchial cryobiopsy, a new semi-invasive method, has been used to obtain adequate specimens in diffuse parenchymal lung disease instead of transbronchial lung biopsy [29], it is possible that transbronchial cryobiopsy may be an alternative to OLB in ARDS patients.

The definition of AIP includes the rapid onset of respiratory symptoms, development of acute respiratory failure with bilateral lung infiltrates, absence of a known cause or predisposing illness, and documentation of DAD on pathology [10]. Patients who fulfill the clinical and pathologic criteria for AIP overlap substantially with those who meet the clinical criteria for ARDS. Of the 64 ARDS patients who conformed to the Berlin definition of ARDS in whom OLB confirmed DAD, 17 with an unknown etiology could be defined as having AIP. One of the features of AIP is acuteness of the onset of respiratory symptoms, which has been reported to range from 2 to 11 days up to 2 months [12,30,31]. Although there is currently no consensus on an explicit time frame in terms of the duration of the onset of AIP, the timing of acute onset of ARDS is defined as within 1 week according to the Berlin definition [3]. This seems to suggest that one subgroup or phenotype of ARDS patients involves those with AIP who present with less acute symptoms, unknown etiology and pathologically confirmed DAD.

There is insufficient evidence to make a definitive recommendation with regards the use of glucocorticoids, despite many clinical trials conducted on ARDS patients [32]. Whereas one study reported no benefit with corticosteroid therapy in patients with influenza A/H1N1-related ARDS [33], another recent retrospective study reported that corticosteroid therapy provided survival benefits in patients with aspiration-related ARDS [34]. In this retrospective study, we investigated the effect of glucocorticoids in a more homogenous group of ARDS patients with pathologically confirmed DAD. The effect of glucocorticoids seemed to have no survival benefits in our ARDS patient with DAD, despite the high rate of glucocorticoid prescriptions (up to 80%). Future prospective studies should expand the current physiological definition of ARDS with biomarkers corresponding to a specific phenotype, such as DAD, to identify steroid-sensitive patients.

There are several limitations to the present study. First, it is a retrospective study conducted at a single referral medical center, which may limit the generalizability of the results to other hospitals. Second, the decision to perform OLB with subsequent DAD findings was highly selective without randomization, and only a small proportion of the patients with ARDS were referred for a biopsy. Finally, it is possible that the number of DAD patients with a known etiology was underestimated, because the identification of microbiology depends on the availability of laboratory facilities.

In summary, the patients with ARDS and DAD confirmed by OLB are a specific phenotype associated with a high mortality rate of 71.9%. Our findings suggest that SOFA score is an independent predictor of hospital mortality in ARDS patients with pathologically confirmed DAD. Glucocorticoid treatment did not seem to improve the morality rate in these patients, however further prospective studies are needed to confirm our findings.
